# Some German Literature of Interest to the Profession

**Published:** 1900-12

**Authors:** William H. Potter

**Affiliations:** Boston, Mass.


### International Dental Journal.

Vol. XXI. December, 1900. No. 12.

### Original Communications.[1]

### SOME GERMAN LITERATURE OF INTEREST TO THE PROFESSION.[2]

BY WILLIAM H. POTTER, D.M.D., BOSTON, MASS.

No one can come in contact with the intellectual life of the German-speaking nations without realizing that it is one of the greatest powers in the mental world. This intellectual life is developed by a thorough primary, secondary, and higher education, and is favored by a capacity for minute and prolonged investigation which seems to be born into the German mind. Hence the literature of such a people must always be of value to the student.

It has commonly been taken for granted that, however much the Germans, or, for that matter, all Europeans, may be looked up to in most branches of learning, in the science of dentistry little or nothing was to be learned from them. Before accepting this supposition as true, we ought to separate the science of dentistry into two divisions,—the theoretical, or intellectual, and the practical, or operative.

While it is true that the object of the science of dentistry is to

[1] The editor and publishers are not responsible for the views of authors of papers published in this department, nor for any claim to novelty, or otherwise, that may be made by them. No papers will be received for this department that have appeared in any other journal published in the country.

[2] Read before the American Academy of Dental Science, October 3, 1900.

produce practical operations, yet practical work is always founded upon theoretical knowledge, and can only advance with the advance of theoretical knowledge. It is not well, then, to look slightingly upon the purely theoretical side of the profession. It seems probable that the practical side of our science has been and probably always will be farther advanced in our own country than in any other place. I am not so sure, however, that we can always claim leadership in matters pertaining to the theoretical or purely intellectual division.

From the observations which I was able last winter to make in Berlin and Vienna, it seemed to me that the men who were being trained for the practice of dentistry in the universities there located were better fitted by thorough preliminary education, and through well-conducted scientific education, to deal with the theoretical or purely scientific part of our profession than we. At the University of Vienna the dental student must graduate in medicine first, and then enter the dental department. He is a thoroughly trained scientific man, competent to undertake the most difficult scientific problems connected with the science of dentistry. His skill in the practical side of dentistry depends upon his own aptitude and inclination. No specified course is required for practical work, and there is little probability that he will acquire the technical skill which is common among the graduates of our best schools.

Let me tell you how I became acquainted with one book which I wish to present to your attention to-night. Wishing to inspect the dental department of the University of Vienna, and having no letters of introduction to men connected with it, I looked up the department in the catalogue of the University. Here I found it represented by five courses given at the "Zahnärztliches Institut," and from the catalogue I decided that Dr. Julius Scheff was the leading man. So one afternoon, just before the time at which this gentleman was put down for a lecture, I presented myself at the dental school and handed in my card. I was soon ushered into the private room of Dr. Scheff and very cordially received by him, after having introduced myself as an American dentist, and as being connected with the dental department of Harvard University. After a few minutes conversation Dr. Scheff introduced me to his assistant, Dr. Rudolf Loos.

Dr. Loos very politely showed me about the operating-rooms so that I could see the students at work, and then invited me into his laboratory. Here he brought out some very interesting sections of the upper and under jaw. These sections were both horizontal and vertical; the vertical sections included the tooth-structure as well as the bony structure, no soft tissues being included in the sections. After showing me the sections, he handed me a book which he had written on the "Anatomical Construction of the under Jaw as a Foundation for the Mechanics of Extraction."[1] And it is this book which I wish to call to your attention first.

I cannot give a better idea of the book than by going over the various heads into which the subject is divided. After a description of the general features of the under jaw there comes a chapter entitled "The Topographical Relation of the Alveoli to the so-called 'Plates' of the Jaw." We next come to "the relations of the alveoli to one another and to the division walls." A chapter on "A Comparison of the Vertical and Cross Sections of the Under Jaw" brings out many interesting anatomical relations of the root at different levels with the spongiosa and the outer wall plates. After the foregoing intimate study of the tissues of the inferior maxillary bone, and the position of the roots of the teeth in these tissues, the subject of the mechanics of extraction is next entered upon. Each tooth is separately considered, and from the anatomical position of the root in reference to the spongiosa and the dense outside plates the direction of least resistance is determined, and the force applied accordingly.

After a consideration of the use of the forceps as an instrument for extraction, Dr. Loos comes to a consideration of the elevator. I feel sure from personal observation that Dr. Loos has a fondness for this kind of instrument, and I have seen him use it with skill.

In concluding my remarks upon this book I wish to call attention to its thoroughness. It is intended as a guide to practical work, and yet in order to teach how to extract a tooth it begins with a minute treatment of the architectural anatomy of the lower jaw. It deals with the relations of hard and soft tissues, and the manner in which each root throughout its entire length is placed in regard to these tissues.

After having thoroughly gone over the foundation knowledge,

---

[1] Der anatomische Bau des Unterkiefers als Grundlage der Extractionsmechanik, von Dr. Rudolf Loos, Assistent am zahnärztlichen Institut der K. K. Universität, Wien.

it then applies it for the purpose in hand. To me the book has its chief interest as a study in anatomy, though its practical side is also important.

The second book which I would call to your attention is one on "The Affections of the Mouth,"[1] by J. Mikulicz and W. Kümmel. This book contains a very complete description of the pathological conditions to be found in the oral cavity. I was especially interested in reading the long chapter devoted to syphilis of the mouth, and the short chapter on the affection called "Lingua geographica," or "Annulus migrans." This affection of the tongue is one which we as practitioners often meet, and I have no doubt often wonder as to its significance. The subject is here very completely treated and an excellent photograph of a case is given.

A third book which I will present to you to-night is upon "The Topographical Anatomy of the Head and Neck,"[2] by Zuckerkandl. Professor Zuckerkandl is one of the principal men in the anatomical deparatment of the University of Vienna, and his book recently published is full of finely executed plates covering the oral cavity and all adjacent parts. Two of the dissections shown in this book I had duplicated, and brought them home with others for the teaching department of the Harvard Dental School.

The same author has also written a valuable book upon "The Anatomy of the Oral Cavity with Special Regard to the Teeth."[3] This work was published in 1891, and is a careful and complete treatment of the subject.

---

### STATE DENTAL EXAMINING BOARDS AND THEIR QUESTIONS.[4]

BY WILLIAM H. TRUEMAN, D.D.S., PHILADELPHIA.

THE advent and the evolution of the State Dental Examining Boards has not proved, either to the profession or to the community, wholly good or thoroughly bad. In so far as they were designed to

---

[1] Die Krankheiten des Mundes, von J. Mikulicz und W. Kümmel, Breslau.

[2] Atlas der topographischen Anatomie des Menschen, von Professor Dr. E. Zuckerkandl.

[3] Anatomie der Mundhöhle mit besonderer Berücksichtigung der Zähne, von Professor Dr. E. Zuckerkandl.

